# Multi-Omic Factors Associated with Frequency of Upper Respiratory Infections in Developing Infants

**DOI:** 10.3390/ijms24020934

**Published:** 2023-01-04

**Authors:** Ramin Beheshti, E. Scott Halstead, Bryan Cusack, Steven D. Hicks

**Affiliations:** Pennsylvania State Health Children’s Hospital, Department of Pediatrics, 500 University Drive, Hershey, PA 17033, USA

**Keywords:** multi-omic, upper respiratory infections, infants, miRNA, viral infections

## Abstract

Susceptibility to upper respiratory infections (URIs) may be influenced by host, microbial, and environmental factors. We hypothesized that multi-omic analyses of molecular factors in infant saliva would identify complex host-environment interactions associated with URI frequency. A cohort study involving 146 infants was used to assess URI frequency in the first year of life. Saliva was collected at 6 months for high-throughput multi-omic measurement of cytokines, microRNAs, transcripts, and microbial RNA. Regression analysis identified environmental (daycare attendance, atmospheric pollution, breastfeeding duration), microbial (*Verrucomicrobia*, *Streptococcus phage),* and host factors (miR-22-5p) associated with URI frequency (*p* < 0.05). These results provide pathophysiologic clues about molecular factors that influence URI susceptibility. Validation of these findings in a larger cohort could one day yield novel approaches to detecting and managing URI susceptibility in infants.

## 1. Introduction

Upper respiratory infections (URIs) are the most common cause of healthcare visits worldwide [1]. URIs are a major cause of chronic respiratory disease exacerbations [1]. Infants are particularly susceptible to URIs and URI-related hospitalization [2]. Enhancing our understanding of how environmental and biologic factors interact to influence URI risk could provide opportunities to develop novel interventions and therapeutics, reducing the burden of URIs on individuals and society.

Infant predisposition to URIs is influenced by environmental risk factors [3]. Infants who attend day care, live in large households, or require recurrent healthcare visits are at risk for experiencing a high number of URIs [4]. Additionally, secondhand smoking exposure, low birth weight, and malnutrition have been associated with increased frequency of URIs in infants [4]. Genetic risk factors have also been identified [5]. Genes affecting interferon (IFN) signaling, interleukins, angiotensin-converting enzymes, and transmembrane serine protease-2 are critical for maturation of the developing immune system [5]. Polymorphisms in immune regulatory genes have been associated with sub-optimal immune responses and elevated risk of infection [6,7]. One mechanism by which infant genetics regulates immune responsivity is through the production and release of cytokines [7].

Cytokines, such as C-X-C Motif Chemokine Ligand 10 (CXCL10), interleukin 18 (IL-18), interleukin 6 (IL-6), and interleukin 8 (IL-8) are involved in the early stages of inflammation associated with viral URIs [8,9]. There is evidence indicating that the risk of acquiring URIs and the risk of developing severe complications are mediated through cytokine activity, which is regulated by immune system genes [10]. Aberrant cytokine production may contribute to increased airway inflammation associated with more severe symptoms [10]. Inter-individual variability in cytokine responses may stem from genetic, epigenetic, or epi-transcriptional factors, such as micro-ribonucleic acids (miRNAs) [11,12].

miRNAs are short, non-coding nucleic acids that modulate protein expression by binding and sequestering messenger RNAs for degradation [13]. Intriguingly, host miRNAs also interact with viral pathogens [14]. These miRNA-virus interactions can promote or inhibit the virus life cycle. Studies have revealed that specific miRNAs are over-expressed in response to viral replication [15], and that miRNAs can down-regulate mRNA targets with protective anti-viral function [15,16]. This down-regulation may permit virus replication, maturation, and release [15,16]. Alternatively, viral gene products may also influence miRNA expression to regulate their own replication, promoting evasion of the host immune response, and increasing viral latency [17]. Therefore, two-way communication between viruses and miRNAs may profoundly impact URI frequency and duration [14,15,16,17].

The microbiome has also been implicated in viral URIs [18]. Changes in the respiratory microbiome have been reported in patients with viral URIs [19]. Similar observations have been reported in infantile wheezing episodes [20]. It remains unclear, however, if changes in the composition of the gut or respiratory microbiota (microbial dysbiosis) predisposes infants to URIs. Given the potential importance of host-microbial interactions in URI pathogenesis, the correlations between oral microbes and the host immune response, and the ability to measure genetic, cytokine, and miRNA factors in the oropharynx, the current study harnessed infant saliva samples to interrogate mult-omic networks with potential involvement in URI pathogenesis.

**Figure 1:**
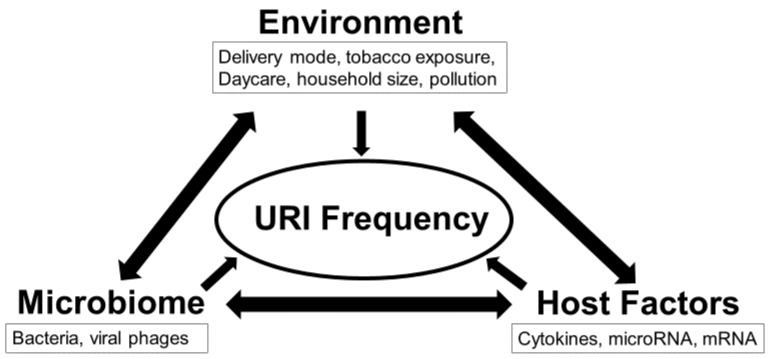
Interactions between environment, microbiome, and host immune factors determine frequency of upper respiratory infection (URI) in the first 12 months. This study utilized a multi-omics approach to test the overarching hypothesis that interactions between the exposome, microbiome, and inflammasome dictate URI frequency.

Although various molecular factors have been implicated in immune system maturation and URI predisposition [14,15,16], to our knowledge, few studies have employed a multi-omic approach to explore their interactions in developing infants. Such information could enhance our knowledge about the pathophysiologic mechanisms underlying URIs, leading to novel approaches for prevention and treatment. This study tested the hypothesis that interactions between specific microbes, miRNAs, mRNAs, and cytokines could modulate URI frequency (Figure 1). This hypothesis was tested using a longitudinal cohort study of 146 infants from birth through 12 months of age.

## 2. Results

### 2.1. Participants

The majority of the 146 participating infants were female (82, 56%), white race (121, 82%), and non-Hispanic (132, 90%; Table 1). The average gestational age at delivery was 39 (±1) weeks, and the average birth weight was 3376 (±445) grams. Most (111, 76%) lacked a family history of asthma. Most infants were delivered vaginally (117, 80%). The majority did not attend daycare (90, 61%), and were not exposed to passive tobacco use (130, 89%), or atmospheric pollution (91, 62%). The average duration of exclusive breastfeeding was 5 months (±2; range = 0–12), and the average household size was 3 persons (±1; range = 2–9).

### 2.2. URI Characteristics

The average number of URIs reported in the first 12 months was 2 (±2; range = 0–12). The first URI episode occurred, on average, 1 (±1) month after birth. No participants were hospitalized for bronchiolitis, two received a diagnosis of pneumonia, two received a prescription for albuterol, and three were prescribed oral steroids.

### 2.3. Multi-Omic Analysis

Step-wise linear regression identified eight omic features that accurately (R^2^ = 0.39, AIC = 593, RMSE = 1.72, *p* < 0.001) modeled the number of URIs in the first 12 months (Table 2).

**Figure 2:**
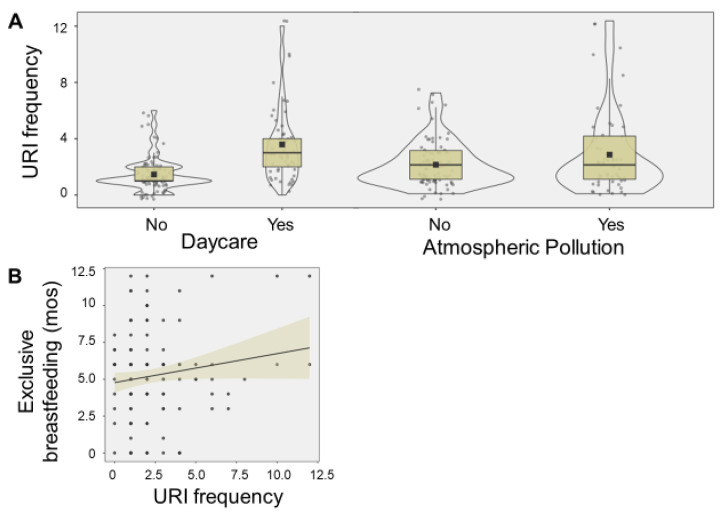
Exposome relationship with URI frequency. The violin plots display number of upper respiratory infections (URIs) associated with daycare attendance (F = 52.8, *p* < 0.001), and atmospheric pollution (F = 7.76, *p* = 0.006) (**A**). The boxes denote 95% confidence intervals, along with mean (square) and median values (black line). The scatter plot display the relationship between number of URIs and the duration of duration (months (mos)) of exclusive breastfeeding (F = 4.27, *p* = 0.041) (**B**). A trend line and 95% confidence interval (shaded area) are displayed.

Higher number of URIs were associated with daycare attendance (F = 52.8, *p* < 0.001), atmospheric pollution (F = 7.76, *p* = 0.006), and longer duration of exclusive breastfeeding (F = 4.27, *p* = 0.041) (Figure 2).

**Figure 3:**
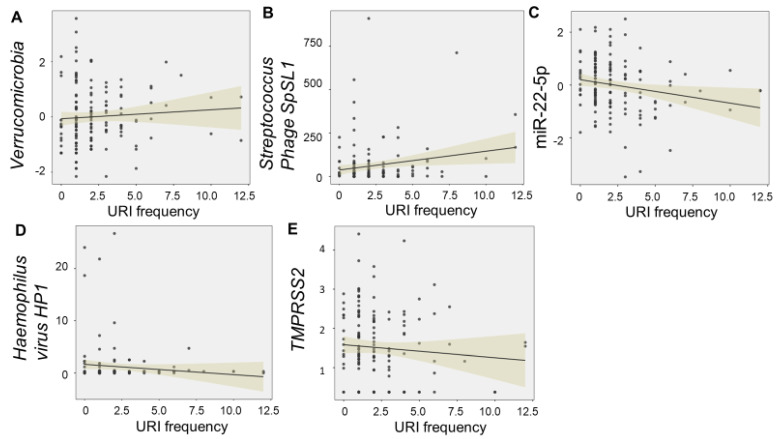
Multi–omic relationships with URI frequency. The scatter plots display significant relationships between frequency of upper respiratory infections (URIs) in the first 12 months and levels of salivary “omic” factors. Levels of *Verrucomicrobia* (F = 7.77, *p* = 0.006) (**A**) and *Streptococcus phage SpSL1* (F = 6.57, *p* = 0.011) (**B**) were directly associated with the number of URIs, whereas levels of miR-22-5p (F = 6.93, *p* = 0.009) (**C**) were inversely related. *Haemophilus virus HP1* (F = 2.56, 0.11) (**D**), and *TMPRSS2* (F = 2.24, *p* = 0.13) (**E**) met criteria for model inclusion (F > 2.0), but did not display significant relationships with URI frequency. Trend lines with 95% confidence intervals are displayed for each relationship.

Salivary levels of *Verrucomicrobia* (F = 7.77, *p* = 0.006) and *Streptococcus phage SpSL1* (F = 6.57, *p* = 0.011) were directly associated with the number of URIs, whereas levels of miR-22-5p (F = 6.93, *p* = 0.009) were inversely related. *Haemophilus virus HP1* (F = 2.56, 0.11), and *TMPRSS2* (F = 2.24, *p* = 0.13) met criteria for model inclusion (F > 2.0), but did not display significant relationships with URI frequency (Figure 3).

**Figure 4:**
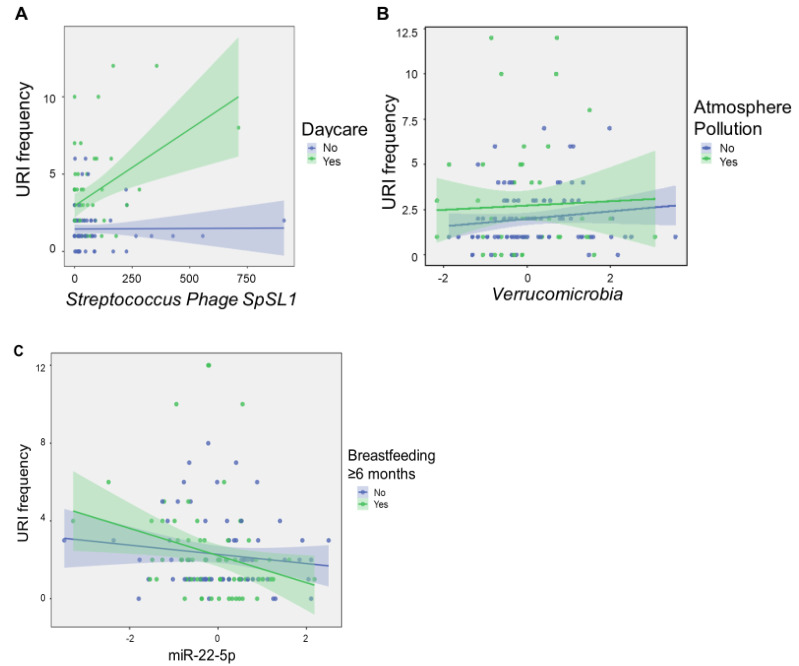
Microbial factors modulate environmental impacts on URI frequency. The scatter plots show molecular features of interest that displayed significant interaction effects with environmental exposures on linear regression analysis. *Streptococcus phage SpSL1* potentiated the number of URIs among infants who attended daycare (F = 12.1, *p* < 0.001) (**A**), whereas *Verrucomicrobia* potentiated URI frequency in infants without atmospheric pollution exposure (F = 3.21, *p* = 0.009) (**B**), and miR-22-5p levels potentiated the protective effects of breastfeeding ≥ 6 months (F = 10.8, *p* = 0.001) (**C**). Normalized, scaled read counts are displayed for *Streptococcus phage SpSL1, Verrucomicrobia,* and miR-22-5p. URI frequency represents the number of URIs reported in the first 12 months after birth. Shaded areas represent 95% confidence intervals.

Notably, no cytokine components met criteria for inclusion in the model, although levels of CXCL10 were modestly correlated with the number of URIs (R = 0.20, *p* = 0.024). There were significant interactions between daycare attendance and levels of *Streptococcus phage SpSL1*, as well as *Verrucomicrobia* levels and atmospheric pollution. *Streptococcus phage SpSL1* potentiated the number of URIs among infants who attended daycare (F = 12.1, *p* < 0.001), whereas *Verrucomicrobia* potentiated URI frequency in infants without atmospheric pollution exposure (F = 3.21, *p* = 0.009; Figure 4). A significant interaction effect was also observed between levels of miR-22-5p and duration of exclusive breastfeeding (F = 10.8, *p* = 0.001). High levels of infant miR-22-5p had a protective effect on URI frequency among infants with longer breastfeeding duration.

## 3. Discussion

In this cohort of 146 infants, we identified three environmental factors (daycare attendance, atmospheric pollution, and duration of exclusive breastfeeding), one microbe (*Verrucomicrobia*), and one viral phage (*Streptococcus phage SpSL1*) that were directly associated with URI frequency in the first 12 months. One miRNA (miR-22-5p) was inversely correlated with URI frequency. Abundance of specific microbes in the infant oropharynx modulated the impacts of environmental exposures on URI frequency.

Several “omic” features in this study have been described in previous studies of viral pathogenesis. For example, miR-22 has been shown to inhibit adenovirus replication within lung fibroblasts by targeting retinoblastoma (Rb) dependent cell cycle genes [21,22]. Rb inactivation may increase pro-inflammatory signaling by stimulating the interleukin-6/STAT3 pathway [23]. In this study miR-22-5p was associated with less frequent URI infections which supports its protective role in suppressing viral replication and proliferation. *TMPRSS2* also aided modeling of URI frequency. Prior studies have found that *TMPRSS2* promotes viral propagation by activating the spike protein and enhancing viral entry into host cells in severe acute respiratory syndrome-related coronavirus (SARS-CoV) and Middle East respiratory syndrome-related coronavirus (MERS-CoV) [24]. It is possible that the infant upper respiratory tract responds to recurrent viral URI infections by down-regulating *TMPRSS2* expression in an effort to reduce URI frequency. Additionally, these immunoregulatory mediators have been demonstrated to influence the pro-inflammatory shifts in influenza A and respiratory syncytial virus (RSV) mediated URIs [25,26].

Host immunologic factors may be driven by microbial flora in the oropharynx [27]. For example, elevated levels of *Haemophilus*, *Corynebacterium*, *Dolosigranulum*, and *Streptococcus* in saliva have been associated with perturbations in host immunity that are linked to activation of innate immune mechanisms involved with URIs and airway remodeling [27,28,29]. One study found that aberrant presence of *Verrucomicrobia* in the gut can induce pro-inflammatory chemokines implicated in neuroinflammation [30]. Bacteriophages, such as *Streptococcus phage SpSL1* may also induce inflammatory perturbations in the oral microbiome [29,31]. In this study, levels of *Verrucomicrobia* and *Streptococcus phage SpSL1* were higher in the saliva of infants with more frequent URIs, supporting their pro-inflammatory role in viral infections.

Intriguingly, *Streptococcus phage SpSL1* potentiated the number of URIs among infants who attended daycare, whereas *Verrucomicrobia* potentiated URI frequency in infants without atmospheric pollution exposure. These results are consistent with prior studies demonstrating that air pollution and daycare exposure may interact with host or microbial factors to influence infection susceptibility [32,33,34]. Exposure to air pollution during infancy has been shown to alter the presence of symbiotic microbes that impact regulatory T cell function in reactive airway disease [31], and cause intestinal microbiome shifts critical to the lung-gut microbiome axis [34]. Daycare exposure may also impact individual microbiome variability [35], which can, in turn, influence host miRNAs involved in host immune responses [36,37,38].

**Figure 5:**
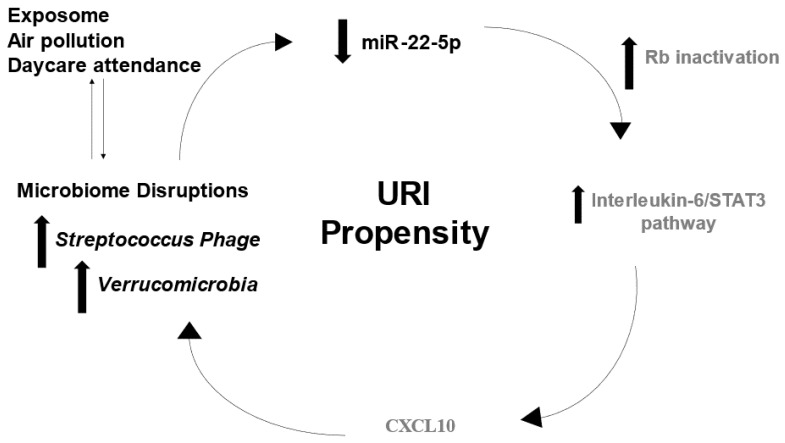
Putative multi-omic mechanism for URI susceptibility. The concept diagram displays mechanisms through which molecular observations in this cohort may contribute to URI propensity. Decreased levels of miR-22-5p lead to retinoblastoma (Rb) inactivation, which may increase pro-inflammatory signaling by stimulating the interleukin-6/STAT3 pathway. Microbial diversity may be influenced by cytokine signaling or environmental exposures. Increased activity of *Verrucomicrobia* and *Streptococcus Phage* could influence epi-transcriptional regulators of the immune response (i.e., miR-22-5p) that increase URI risk. Black text denotes factors identified in this cohort, whereas gray text denotes interactions reported in the scientific literature.

Based on the findings of this study, we propose a conceptual framework for URI pathogenesis involving microbial perturbations, miR-22-5p expression, immune dysregulation, and environmental exposures (Figure 5). In this conceptual model, miR-22 inhibits viral replication through inactivation of Rb and enhancement of pro-inflammatory signaling (i.e., interleukin-6/STAT3 pathway) [23]. Cytokine shifts may perturb microbial diversity through complex interactions between lipopolysaccharide (LPS), Toll-Like Receptor 4 (TLR4), TMPRSS2, and miRNA regulation [21,22,23,24,27,28,29,30,31]. Environmental exposures such as daycare attendance and air pollution may also influence microbial composition and blunt immune responses that predispose infants to frequent URIs [32,33,34,35]. Intriguingly, miR-22-5p levels appear to potentiate the protective effect of sustained breastfeeding. This is consistent with prior studies suggesting that maternal-infant transfer of miRNAs in breastmilk may impact immune development [39]. It also supports prior studies implicating miR-22 expression in upper respiratory infections, pneumonia, and asthma [40,41].

There are several limitations of this study. URIs were identified through validated surveys and review of the medical record. However, surveys could introduce recall bias, and it is possible that the medical record might miss URIs that were diagnosed at an outside facility. We do note that participants were followed at our outpatient pediatric clinic for routine well-child check-ups, at which time recent urgent care or outpatient visits were typically recorded in the medical record. Although the vast majority of infant URIs are viral in nature, the precise pathogen responsible for each infection episode was not confirmed through laboratory testing. It is possible that some infectious episodes involved bacterial pathogens. Finally, the primary outcome of the study was URI frequency. Although details regarding severity were recorded, the study was not powered to compare multi-omic profiles between infants with severe and non-severe URI episodes. This should be explored in future studies.

The results of this study support the premise that multi-omic analysis of infant saliva may provide insight into pathophysiologic mechanisms that underlie URIs. Such knowledge could eventually aid in novel diagnostic, preventative, or therapeutic measures. Validation of these results in a larger cohort with ancestral and geographic diversity is required.

## 4. Materials and Methods

### 4.1. Ethics

This study was approved by the Institutional Review Board at the Penn State College of Medicine (STUDY00008657). Parents/guardians provided written, informed consent.

### 4.2. Participants

**Figure 6:**
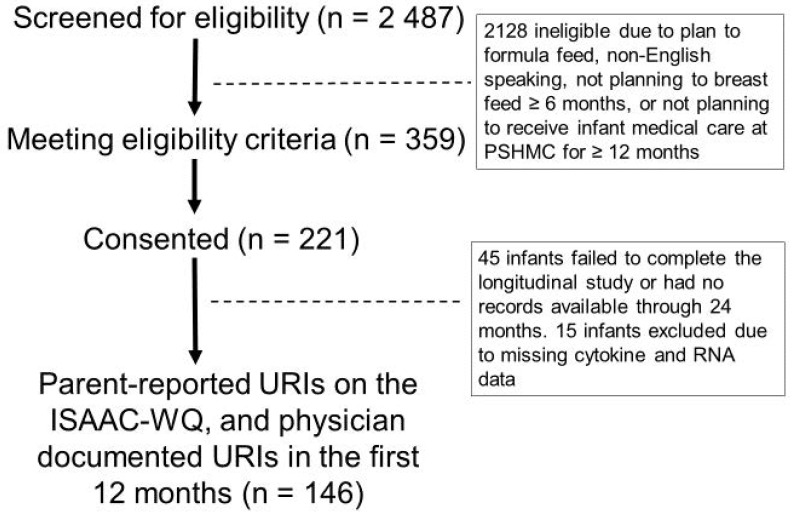
Participant CONSORT diagram. The CONSORT diagram displays the number of participants who were screened (2487), eligible (359), consented (221), and completed the 12-month study (146).

This study involved a subset of infants from a prospective longitudinal cohort of 221 mother-infant dyads [39]. Eligibility criteria were term delivery (37–42 weeks gestation) and plan to breastfeed ≥ four months. Exclusion criteria were factors affecting long-term follow up (e.g., plan for primary pediatric care at an outside hospital, plan for infant adoption), inability to complete electronic surveys (e.g., non-English speaking), and medical conditions that could impact breastfeeding ability (e.g., maternal drug addiction, human immunodeficiency virus infection, infant cleft lip/palate, NICU admission > 7 days). A convenience sample was enrolled within seven days of delivery. Enrollment occurred between 20 April 2018 and 5 October 2020 at the newborn nursery or the outpatient pediatrics clinics affiliated with our academic medical center. Among the 2487 infants screened for eligibility, there were 359 who met criteria, and 221 consented to participate. Of these, 146 completed the study (Figure 6). The primary medical outcome was number of URIs in the first 12 months after birth, as reported by parents/guardians on the International Study of Asthma and Allergies in Childhood–Wheezing Questionnaire (ISAAC-WQ) [42]. Review of medical records by research staff was used to confirm presence/absence of acute appointments for URI symptoms for all participants.

### 4.3. Survey Collection

Electronic surveys administered by research staff were used to obtain medical and demographic characteristics at the time of enrollment. Survey responses were confirmed through review of the electronic medical record. The following medical and demographic characteristics were collected: biological sex, race, ethnicity, gestational age (weeks), birth weight (g), and family history of asthma. The National Survey of Lead and Allergens in Housing (NSLAH) [43] was administered at 1 month of age to assess the infant “exposome”: delivery mode (vaginal, cesarean section), passive tobacco exposure, duration of exclusive breastfeeding (months), daycare attendance, number of people living in the household, and atmospheric pollution. Details about URIs were collected using the ISAAC-WQ, administered at 12 months: age of first upper respiratory infection, hospitalization for bronchiolitis, diagnosis of pneumonia, prescription for albuterol, prescription of oral steroids.

### 4.4. Saliva Collection

Two non-fasting saliva samples were collected from each infant at 6 months of age. One swab contained nucleic acid stabilizer (DNA Genotek, Ottowa, ON, Canada), and was used for analysis of the miRnome (miRNAs), the transcriptome (mRNAs), the microbiome (microbial RNAs), and the virome (viral RNAs). The second swab contained no stabilizing reagents (Oasis Diagnostics, Vancouver, WA, USA), and was used for analysis of the inflammasome (cytokines). Saliva was collected from the sub-lingual and parotid regions by swabbing the oropharynx for 10–20 s. Samples were stored at −80 ℃ prior to molecular analysis.

### 4.5. Inflammasome Analysis

Cytokines were measured using the ELLA SimplePlex immunoassays (Protein Simple, San Jose, CA, USA). The ELLA instrument performs automated, fluorescent-labeled antibody detection of cytokines using a microfluidic cartridge. Immunoassays were used to measure levels of four salivary cytokines: C-X-C Motif Chemokine Ligand 10 (CXCL10), interleukin 18 (IL-18), interleukin 6 (IL-6), and interleukin 8 (IL-8). These cytokines were selected based on their bioavailability in saliva, as well as their relevance to inflammation and URIs [44,45,46,47,48]. As we have previously described, saliva samples were centrifuged for 4 min at 16,000× *g*, and 70 µL of sample was mixed with 70 µL of diluent [49]. The solutions were centrifuged for 2 min at 1000× *g*, then 50 µL of diluted samples were loaded on a custom cartridge and processed on an ELLA Simplex instrument per manufacturer instructions.

### 4.6. RNA Analysis

RNA was extracted from each sample using the miRNeasy Kit (Qiagen, Inc., Germantown, MD, USA), and RNA quality was assessed with an Agilent Bioanalyzer 2100 (Agilent, Santa Clara, CA, USA). Downstream RNA processing was performed as we have previously described [50]. Briefly, RNA sequencing was completed at the SUNY Molecular Analysis Core using the Illumina TruSeq Small RNA Prep protocol and a NextSeq500 instrument (Illumina; San Diego, CA, USA) at a targeted depth of ten million, 50 base, paired-end reads per sample. Reads were aligned to the hg38 build of the human genome using Partek Flow (Partek; St. Louis, MO, USA) and the Bowtie2 aligner. Quantification of messenger RNAs (mRNAs) was performed using Ensembl annotation, and quantification of mature microRNAs was performed using miRBase 22 annotation. RNA reads that were unaligned to the human genome were aligned to the NCBI RefSeq genome using Kraken. Bacteria were aligned at the phylum level and viral phages were aligned at the species level. We chose to measure microbial RNA in order to streamline the nucleic acid extraction and analyses steps. Quality of RNA sequencing results was verified through read quality score and total read count. The RNA features with consistent detection (raw read counts ≥10 in ≥100% of samples) in each category (mRNAs, miRNAs, bacteria, virus) were quantile normalized and scaled prior to statistical analysis. Downstream analysis involved 8 transcripts (*TMPRSS2*, *IL1RN*, *IL6ST*, *CCL2*, *TNFAIP3*, *TLR4*, *TAB2*, *NFKBIA*), 8 miRNAs (miR-140-3p, miR-22-5p, miR-29c-5p, miR-34c-5p, miR-125a-5p, miR-27b-3p, miR-203a-3p, miR-155-5p), 8 viral phages (*Streptococcus phage SpSL1*, *Pseudomonas phage PPpW-3*, *Proteus virus PM135*, *Streptococcus phage phiARI0131-1*, *Mycobacterium virus Cooper*, *Bacillus virus Mater*, *Haemophilus virus HP1*, *Klebsiella phage vB_KpV477*), and 8 microbial phyla (*Actinobacteria*, *Bacteroidetes*, *Candidatus Saccharibacteria*, *Firmicutes*, *Fusobacteria*, *Proteobacteria*, *Verrucomicrobia*), plus a measure of microbial diversity (Simpson alpha index). These features were selected based on their biologic relevance to viral upper respiratory infections and their abundance in infant saliva [25,26,51,52,53,54]. A maximum of 8 features were permitted in each category based on an a priori power analysis demonstrating that the sample size (*n* = 146) provided > 95% power to detect an effect size ≥ 2.0 for 8 predictors, with alpha set at 0.05 (assuming a non-central distribution).

### 4.7. Statistical Analysis

Descriptive statistics were used to summarize the medical and demographic characteristics of participating infants. The relationship between the primary medical outcome (number of URIs in the first 12 months) and molecular features from the 6 “omic” categories (exposome, inflammasome, transcriptome, miRnome, microbiome, and virome) were assess using linear regression. Number of URIs was the dependent variable, and omic features served as independent variables. Variables were introduced in a feed-forward, step-wise procedure. Only features with F ≥ 2.0 were retained, and a maximum of 8 features was permitted in the final model. For variables with large effect sizes (F > 5.0), interactions were explored. Overall model statistics (Akaike Information Criteria (AIC), R^2^, and Root-mean-square-deviation (RMSE)) and individual factor statistics (ANOVA Omnibus F-value, *p*-value, estimate) were reported. A Durbin-Watson test for autocorrelation was performed, and Q-Q plots were used to visualize residuals. All analyses were completed using Jamovi Software (v2.3) [55].

## 5. Conclusions

The findings in this study suggest that multi-omic analysis of infant saliva may provide insight into pathophysiologic mechanisms that underlie upper respiratory infections, especially interactions between host immunity, microbial activity, and epi-transcriptomic. The measurement of these multi-omic factors may aid the identification of infants at risk for future respiratory infections, allowing clinicians to provide personalized anticipatory guidance and treatment. If a sufficiently accurate tool were developed, the results might be used to guide preventative measures, which could decrease emergency visits and the need for hospitalizations due to severe upper respiratory infections.

## Figures and Tables

**Table 1:** Participant Characteristics.

Characteristic	*n* (%)
All Infants	146 (100)
*Infant Traits*	
Female sex	82 (56)
Gestational age, weeks; mean (SD)	39 (1)
Non-Hispanic Ethnicity	132 (90)
White Race	121 (82)
Birth weight, g; mean (SD)	3376 (445)
Family history of Asthma	35 (24)
*Environmental Exposures*	
Vaginal delivery	117 (80)
Daycare attendance	56 (38)
Tobacco use	16 (11)
Atmospheric pollution	55 (37)
Exclusive breastfeeding, months; mean (SD)	5 (2)
People in household, # (range)	3 (2–9)
*Salivary Factors*	
Age of collection, days; mean (SD)	196 (14)
Time of collection, 24 h; mean (SD)	12 (2)

For discrete characteristics, the table displays the number of participants (*n*) and percent of participants (%) with a given trait. For continuous variables, the cohort mean value and standard deviation (SD) are displayed, with the exception of household size, for which # and range are provided.

**Table 2:** Linear regression utilizing environmental and multi-omic factors models the number of upper respiratory infections in the first 12 months.

			95% Confidence Interval			
Predictor	Estimate	SE	Lower	Upper	t	*p*
Intercept	0.76	0.47	−0.17	1.7	1.6	0.11
Daycare:						
Yes—No	2.3	0.31	1.6	2.8	7.3	< 0.001
Atmospheric Pollution:						
Yes—No	0.86	0.31	0.25	1.5	2.8	0.006
Exclusive breastfeeding (months)	0.11	0.055	0.005	0.22	2.1	0.041
*Verrucomicrobia*	0.42	0.15	0.12	0.73	2.8	0.006
miR-22-5p	−0.42	0.16	−0.74	−0.11	−2.6	0.009
*Streptococcus phage SpSL1*	0.003	0.001	7.08 × 10^−4^	0.005	2.6	0.011
*Haemophilus virus HP1*	−0.06	0.04	−0.14	0.012	−1.6	0.11
*TMPRSS2*	−0.25	0.16	−0.57	0.079	−1.5	0.13

A linear regression utilizing a step-wise, feed-forward procedure was used to identify environmental and omics factors associated with number of upper respiratory infections in the first 12 months after birth. A Durbin-Watson test for autocorrelation was performed, and Q-Q plots were used to visualize residuals.

## Data Availability

FASTQ files from RNA sequencing involved in this project have been deposited into the NIH GEO database (GSE192543). GEO respository link: https://www.ncbi.nlm.nih.gov/geo/query/acc.cgi?acc=GSE192543 (accessed on 25 December 2022).
